# The receptor tyrosine kinase EphB4 is overexpressed in ovarian cancer, provides survival signals and predicts poor outcome

**DOI:** 10.1038/sj.bjc.6603642

**Published:** 2007-03-13

**Authors:** S R Kumar, R Masood, W A Spannuth, J Singh, J Scehnet, G Kleiber, N Jennings, M Deavers, V Krasnoperov, L Dubeau, F A Weaver, A K Sood, P S Gill

**Affiliations:** 1 Department of Surgery, University of Southern California, Los Angeles, CA, USA; 2 Department of Pathology, University of Southern California, Los Angeles, CA, USA; 3 Department of Gynecologic Oncology, University of Texas MD Anderson Cancer Center, Houston, TX, USA; 4 Department of Medicine, University of Southern California, Los Angeles, CA, USA; 5 Department of Pathology and Laboratory Medicine, the University of Texas MD Anderson Cancer Center, Houston, TX, USA; 6 VasGene Therapeutics Inc., Los Angeles, CA, USA; 7 Department of Cancer Biology, the University of Texas MD Anderson Cancer Center, Houston, TX, USA

**Keywords:** EphB4, ovarian cancer, apoptosis, angiogenesis, survival

## Abstract

EphB4 is a member of the largest family of transmembrane receptor tyrosine kinases and plays critical roles in axonal pathfinding and blood vessel maturation. We wanted to determine the biological role of EphB4 in ovarian cancer. We studied the expression of EphB4 in seven normal ovarian specimens and 85 invasive ovarian carcinomas by immunohistochemistry. EphB4 expression was largely absent in normal ovarian surface epithelium, but was expressed in 86% of ovarian cancers. EphB4 expression was significantly associated with advanced stage of disease and the presence of ascites. Overexpression of EphB4 predicted poor survival in both univariate and multivariate analyses. We also studied the biological significance of EphB4 expression in ovarian tumour cells lines *in vitro* and *in vivo*. All five malignant ovarian tumour cell lines tested expressed higher levels of EphB4 compared with the two benign cell lines. Treatment of malignant, but not benign, ovarian tumour cell lines with progesterone, but not oestrogen, led to a 90% reduction in EphB4 levels that was associated with 50% reduction in cell survival. Inhibition of EphB4 expression by specific siRNA or antisense oligonucleotides significantly inhibited tumour cell viability by inducing apoptosis via activation of caspase-8, and also inhibited tumour cell invasion and migration. Furthermore, EphB4 antisense significantly inhibited growth of ovarian tumour xenografts and tumour microvasculature *in vivo*. Inhibition of EphB4 may hence have prognostic and therapeutic utility in ovarian carcinoma.

Ovarian cancer is the second most common gynecologic cancer in women, with an estimated 22 220 new cases in the United States in the year 2005 (American Cancer Society, 2005). Ovarian cancer causes more deaths than any other cancer of the female reproductive system. The vast majority of ovarian cancers is epithelial in origin. Ovarian cancer has a higher incidence in women who carry *BRCA* or mismatch repair gene mutations (Matias-Guiu and Prat, 1998). The role of female sex hormones on the progression of ovarian cancer has also been studied. It is believed that certain metabolites of oestrogen may support tumour growth, whereas progesterone may have a protective role (Seeger and Mueck, 2006). No effective screening tool exists for ovarian cancer and in over 80% cases, the diagnosis is not made until the disease is advanced, making treatment particularly challenging. The current overall 5-year survival is 44%; however, the relative 5-year survival for women with distant disease is only 29% (American Cancer Society, 2005). There is hence a need to identify new molecular markers that play a role in the pathogenesis of ovarian cancer with a hope to offer novel, targeted, biological therapy.

Receptor tyrosine kinases are a diverse group of transmembrane proteins involved in signal transduction pathways that control cell proliferation, differentiation, and migration. The Eph receptors are the largest family of tyrosine kinases, comprising of 15 individual members, divided into EphA and EphB classes. Recently, several studies have documented high expression of the Eph family of receptor tyrosine kinases in tumours (Stephenson *et al*, 2001; Berclaz *et al*, 2003; Wu *et al*, 2004, 2006; Lee *et al*, 2005; Xia *et al*, 2005, 2006; Kumar *et al*, 2006; Masood *et al*, 2006). Although there is limited data on the protein levels of EphB4 in cancers, only recently, data on the biological significance of this protein in tumour biology is being accrued. Evidence for a potential role as a tumour promoter comes from EphB4/neuT transgenic mice, which develop tumours more rapidly than neuT transgenic mice (Munarini *et al*, 2002).

EphB4 and its ligand EphrinB2 show complementary expression on venous and arterial endothelial cells, respectively (Wang *et al*, 1998), and play a critical role in vessel maturation. Knockout of either protein is embryonically lethal in mice owing to vascular arrest at the primitive capillary plexus stage (Gerety *et al*, 1999). Therefore, overexpression of EphB4 by cancers can likely provide an angiogenic advantage to them. However, there is growing evidence that EphB4 may provide more direct survival cues to cancer cells. We have shown recently such a role of EphB4 in prostate (Xia *et al*, 2005), bladder (Xia *et al*, 2006), breast (Kumar *et al*, 2006), and head and neck cancers (Masood *et al*, 2006). In this report, we extend those findings to ovarian cancer as well. We demonstrate a high level of expression of EphB4 in human ovarian tumours, which correlates with advanced stage and short survival. Furthermore, all five ovarian carcinoma cell lines express EphB4. Overexpressed EphB4 provides survival advantage to tumour cells by inhibiting cell membrane originating (extrinsic) apoptosis, and affords an invasive phenotype by promoting tumour cell invasion. EphB4 knockdown effectively reverses these phenomena and inhibits tumour growth in a murine xenograft model of ovarian cancer. EphB4 is thus a novel ovarian tumour marker and a viable target for biological therapy.

## MATERIALS AND METHODS

### Reagents

EphrinB2 (P20) antibody was purchased from Santa Cruz Biotech (Santa Cruz, CA, USA). The anti-phospho-tyrosine antibody 4G10 was from Upstate (Lake Placid, NY, USA), monoclonal anti-actin antibody from Sigma Chemical Co (St Louis, MO, USA), anti-CD31 (M20) from Santa Cruz Biotech (Santa Cruz, CA, USA), monoclonal anti-Ki-67 from DAKO (Carpentaria, CA, USA), and anti-human Fc from Jackson Labs (Bar Harbor, ME, USA). EphrinB2/Fc chimeric protein was from R&D Systems Inc. (Minneapolis, MN, USA). All antisense ODNs and siRNAs were synthesised from Qiagen (Valencia, CA, USA).

Monoclonal antibodies to the extracellular domain of EphB4 were generated in-house at Vasgene Therapeutics (three different clones designated MAb131, MAb47, and MAb265). These antibodies have been characterized extensively in-house and are extremely sensitive and specific for the extracellular domain of EphB4 receptor. They do not cross react with other members of the Eph family of receptors (data not shown). MAb265 detects only denatured human EphB4 on Western blot, MAb131 detects human EphB4 on tissue sections, whereas MAb47 detects human and murine EphB4 and is very effective in immunoprecipitation experiments.

### Cell culture

ML5 and ML10 cells were derived from human ovarian cystadenomas and transfected with SV40 large T antigen to increase their longevity *in vitro*. MCV 50 cells were derived from a subclone of ML10 that became spontaneously immortalized in culture. HOC-7 ovarian carcinoma cells were obtained from Dr R Buick (University of Toronto). OVCAR-3 cells were purchased from ATCC (ATCC#HTB161). ML5, ML10, and MCV50 were cultured in MEM medium supplemented with 10% FCS, 1 mM glutamine, and 1% penicillin–streptomycin (Invitrogen, Carlsbad, CA, USA). HOC-7 and OVCAR-3 cells were maintained in RPMI medium supplemented with 10% FCS, 1 mM glutamine, and 1% penicillin–streptomycin. Hey and CAOV-3 cells were cultured in DMEM medium containing 10% FCS, 1 mM glutamine, 1 mM MEM sodium pyruvate, and 1% penicillin–streptomycin.

### EphB4 siRNAs and antisense oligodeoxynucleotides

EphB4-specific siRNA and phosphorothioate-modified antisense or scrambled oligodeoxynucleotide (ODN) were synthesised (Qiagen, Valencia, CA, USA) and tested for specific inhibition of the EphB4 expression. EphB4-specific siRNA corresponded to the sequence 5′-GGU GAA UGU CAA GAC GCU GUU-3′ and 3′-UUC CAC UUA CAG UUC UGC GAC-5′. The specific siRNA was mutated at three sites to yield siRNAΔ, 5′-**A**GU **U**AA U**A**U CAA GAC GCU GUU-3′ and 3′-UUU CAA UUA UAG UUC UGC GAC-5′, which had no effect on EphB4 levels and was used as control. SiRNA directed against GFP was used as an additional negative control. The AS-ODN used, AS-10, spanned nucleotides 1980–1999 with a sequence 5′-ATG GAG GCC TCG CTC AGA AA-3′. To negate nonspecific cytokine-mediated effects from the CpG site, the cytosine in position 11 was methylated (AS-10M) without loss in EphB4 knockdown effect (data not shown). Scrambled ODN (sequence 5′-AAG GGC TAG GAT AGA CCC TC-3′) with the same nucleotides in a random sequence containing a CpG site as well was used as control.

### Immunohistochemistry

All of the human samples were collected in compliance with requirements of the Institutional Review Board for the Protection of Human Subjects. Formalin-fixed, paraffin-embedded samples were sectioned at 5 *μ*m. Sections were treated with antigen retrieval buffer (modified citrate buffer with DIVA decloaker, BioCare Medical, Concord, CA, USA). Specifically primary monoclonal EphB4 antibody MAb131 was applied overnight at room temperature at a concentration of 30 *μ*g ml^−1^ in 1% BSA/TBST. Slides were incubated in secondary antibody. Immunostaining was carried out using standard techniques (details available on request).

All of the samples were reviewed by a board-certified pathologist who was blinded to clinical outcome of the patients. EphB4 expression was determined by assessing the percentage of stained tumour cells and staining intensity. The percentage of positive cells was scored as follows: 0 points, 0–5%; 2 points, 6–50%; 3 points, >50%. The staining intensity was scored as follows: 1 point, weak intensity; 2 points, moderate intensity; 3 points, strong intensity. Points for expression and percentage of positive cells were added and an overall score between 0 and 6 was assigned. Tumours were categorized into four groups: negative (overall score=0); weak expression (overall score=1–2); moderate expression (overall score=3–4); and strong expression (overall score=5–6). For xenograft staining, sections were incubated with primary antibody (CD31 1 : 250 dilution, Ki-67 1 : 100 dilution) overnight at 4°C. Routine negative controls included deletion of primary and secondary antibody and substitution of normal IgG isotope for primary antibody. When using mouse-anti-human Ki-67 antibody, MOM kit (Vector Labs, Burlingame, CA, USA) was used to block nonspecific binding to mouse tissue. Number of cells staining positive was counted by a blinded observer in five random high-power fields.

### Western blot

Cell lysates were prepared as described by Masood *et al*, (2003). Typically, 10 *μ*g proteins from whole-cell lysate were fractionated on a 4–20% Tris–glycine polyacrylamide gel, electro-transferred to PVDF membrane, and probed with primary antibody overnight. Blot was stripped with Restore™ Western blot stripping buffer (Pierce, Rockford, IL, USA) and reprobed with *β*-actin to confirm equivalent loading and transfer of protein. Signal was detected using SuperSignal West Femto Maximum Sensitivity Substrate (Pierce), quantitated by X-ray densitometry using Fluro-S multi-Imager system (Bio-Rad).

### Phosphorylation analysis of EphB4

Recombinant EphrinB2/Fc or Fc proteins were clustered using anti-Fc antibody for 1 h at 4°C. Hey and Hoc-7 cells were grown in 60 mm dishes until 100% confluence and were treated with clustered EphrinB2/Fc or Fc (Jackson Labs) for various durations of time. Lysates were prepared with buffer containing 20 mM Tris–HCl, pH 8.0, 150 mM NaCl, 1% (v/v) Triton-X100, 1 mM EDTA, 1 mM PMSF, 1 mM sodium vanadate, and centrifuged at 50 000**g** for 60 min at 4°C. Clarified protein samples were incubated overnight with protein A/G coupled agarose beads pre-coated with anti-EphB4 monoclonal antibody (#47). The immunoprecipitated complexes were probed with anti-*p*-Tyr-specific antibody 4G10. EphB4 precipitation efficiency was tested by probing with EphB4 specific monoclonal antibody (#265).

### Wound-healing migration assay

Hey cells were seeded onto six-well plates and cultured until confluent. EphB4 antisense or scrambled ODN (1 *μ*M) or siRNA were introduced to the wells as described for the viability assay. Cell monolayer was wounded by scraping with a sterile pipette tip. Migration of the cells into scraped area over time was examined and recorded with a Nikon Coolpix 5000 digital camera.

### Cell migration assay

Chemotaxis of ovarian cells was assessed using a modified Boyden chamber containing cell culture inserts with an 8 *μ*m pore size Matrigel-coated polycarbonate membrane in 24-well plates (BD Biosciences). The cell suspensions of Hey (2 × 10^5^ cells ml^−1^) in 200 *μ*l DMEM/1%FCS were seeded in the upper chamber following siRNA introduction or along with EphB4 AS-10. Five hundred microlitres of DMEM/1%FCS containing chemotaxis agent EGF (20 ng ml^−1^) was added to the lower chamber. After incubation for 9 h at 37°C, the upper surface of the filter was scraped with swab and filters were fixed and stained with Diff Quick (vWR, West Chester, PA, USA).

### Cell viability assay

Hey and Hoc-7 cells were seeded on 48-well plates at a density of approximately 1 × 10^4^ cells well^−1^ in 200 *μ*l medium. Cells were treated with various concentrations (1–10 *μ*M) of EphB4 AS-10 or scrambled ODN on day 1 and day 3 after cell seeding. After 3 days, medium was changed and fresh ODNs added. Cell viability was assessed by MTT as described previously (Masood *et al*, 2003). EphB4 siRNAs (10–100 nM) were introduced into 2 × 10^4^ cells well^−1^ of a 48-well plate using 2 *μ*l of Lipofectamine™ 2000 according to the manufacturer's instructions. Four-hour post-transfection, the cells were returned to growth medium. Viability was assayed after 48 h as described by Masood *et al* (2003).

### Cell-cycle analysis

Eighty per cent confluent cultures of Hey cells in six-well plates were transfected with various siRNAs (100 nM) using Lipofectamine™ 2000 for variable amount of time, cells were trypsinized, washed in PBS, and incubated for 1 h at 4°C in 1 ml of hypotonic solution containing 50 *μ*gml^−1^ propidium iodide, 0.1% sodium citrate, 0.1 Triton X-100, and 20 *μ*gml^−1^ DNase-free RNaseA. Cells were analysed in linear mode by flow cytometry. Results are expressed as percentages of elements detected in the different phases of the cell cycle, namely sub-G_0_ peak (apoptosis), G_0_/G_1_ (no DNA synthesis), S (active DNA synthesis), G_2_ (premitosis) and M (mitosis).

### Apoptosis assay

Apoptosis was studied using the Cell Death Detection ELISA plus Kit that detects cytoplasmic nucleosomes according to the manufacturer's instructions (Roche, Piscataway, NJ, USA). Briefly, cell in 24-well plates cultured to 80% confluence were transfected using Lipofectamine™ 2000 with various concentrations (0–100 nM) of EphB4 siRNA(472) or GFP siRNA. After 16 h, cells were lysed and nuclei pelleted, incubated with anti-histone-biotin and anti-DNA POD in streptavidin-coated 96-well plate. Colour was developed with ABST and absorbance at 405 nm was read in a microplate reader (Molecular Devices, Sunnyvale, CA, USA). Caspase-8 and -9 activity was measured using the caspase-8 and caspase-9 colorimetric assay kits that monitor cleavage of caspase-8 and -9 peptide substrates (R&D Systems Inc., Minneapolis, MN, USA). Apoptosis was detected in deparafinised sections of animal tumours by TUNEL assay using the *in situ* cell-death detection kit (Roche, Piscataway, NJ, USA) according to the manufacturer's instructions.

### Murine tumour xenograft model

Hey cells were propagated, collected by trypsin digestion, and re-suspended in serum-free medium. 2 × 10^6^ cells were injected in the flank of 10- to 12-week-old female Balb/C athymic mice. Tumour growth was measured three times a week and volume estimated as 0.52 × *a* × *b*^2^, where *a* and *b* are the largest and smallest lengths of the palpable tumour. On day 4 after cell implantation, tumour volumes were calculated to ensure uniformity in size and animals were divided randomly into three groups (*n*=6 mice per group). Each group was administered daily by intraperitoneal injection, AS-10 or scrambled ODN at a dose of 10 mg kg^−1^ or vehicle alone (sterile normal saline, pH 7.4). Animals were killed and tumours and normal organs harvested after 4 weeks. A portion of the tumours was fixed in formalin for paraffin-embedding and histologic analysis. The remaining tumour tissue and organs in each group were pooled and protein was extracted. All procedures were approved by our Institutional Animal Care and Use Committee and performed in accordance with the Animal Welfare Act regulations.

### Statistical analysis

The *χ*^2^ test was used to determine differences among variables using SPSS (SPSS Inc., Chicago, IL, USA). Survival curves were generated with the Kaplan–Meier method and compared with the log-rank statistic. The Cox proportional hazards model was used for multivariate analysis. Student's *t*-test was used to compare tumour volumes. A *P*<0.05 was considered statistically significant.

## RESULTS

### EphB4 expression in human ovarian tumour samples

**Figure 1:**
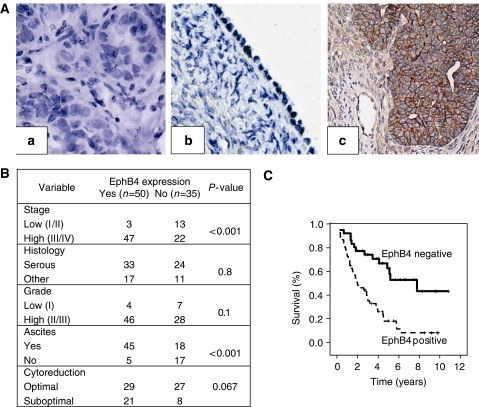
EphB4 is expressed in human ovarian tumour specimens and correlates with advanced stage, presence of ascites, and poor survival. (**A**) Representative immunohistochemical peroxidase staining for EphB4 in a negative (deletion of primary antibody) control (a), normal ovarian epithelium (b), and invasive ovarian carcinoma (c). All pictures were taken at original magnification of × 200. (**B**) Correlation of clinical and pathological variables with EphB4 overexpression in ovarian carcinoma. (**C**) Kaplan–Meier survival of patients with invasive ovarian cancer based on EphB4 staining intensity, using the log-rank statistic.

EphB4 expression in human ovarian samples was assessed by immunohistochemical staining of sections isolated from seven normal ovaries and 85 invasive epithelial ovarian cancers (Figure 1A). All of the normal ovaries had little or absent EphB4 expression on the surface epithelium (Figure 1Ab). Among the invasive ovarian cancers, 73 (86%) expressed EphB4 and moderate or strong expression was noted in 49 (58%) samples. We confirmed EphB4 expression by Western blot in a select group of 10 ovarian tumour samples for which frozen tissues were available. Seven of 10 tumour specimens expressed EphB4 on Western blotting (data not shown), confirming the staining data.

### EphB4 expression correlates with clinicopathological features

The demographic features of patients with invasive ovarian cancer are summarised in Figure 1B. Mean age at presentation was 59.4 (range=34–86) years. Sixty-nine of 85 patients (81%) had advanced stage (III or IV) disease, and 74 of 85 patients (87%) had high-grade (II or III) disease. Fifty-six out of 85 patients (66%) underwent optimal surgical cytoreduction (<1 cm residual disease at completion of surgery). There was no association between expression of EphB4 and histological subtype, tumour grade or extent of cytoreduction.

EphB4 overexpression correlated with advanced disease stage. High expression of EphB4 was detected in 69% of high-stage ovarian cancers compared with 19% of low-stage ovarian cancer (*P*<0.001). Ninety per cent of patients with EphB4 overexpression had ascites compared with 49% of patients without EphB4 overexpression (*P*<0.001). Survival rates of patients with invasive ovarian cancer and EphB4 expression were determined using the Kaplan–Meier method (Figure 1C). In univariate analysis, EphB4 overexpression was associated with significantly worse survival compared with low expression (median survival=7.75 years *vs* 2.58 years; *P*<0.001). On multiple regression analyses using a Cox proportional hazards model that included EphB4 expression, stage, grade, ascites, histology, and level of cytoreduction, only advanced stage (*P*=0.04), EphB4 overexpression (*P*=0.003), and suboptimal cytoreduction (*P*=0.017) were significant predictors of poor survival.

### EphB4 receptor is highly expressed in ovarian carcinoma cell lines

**Figure 2:**
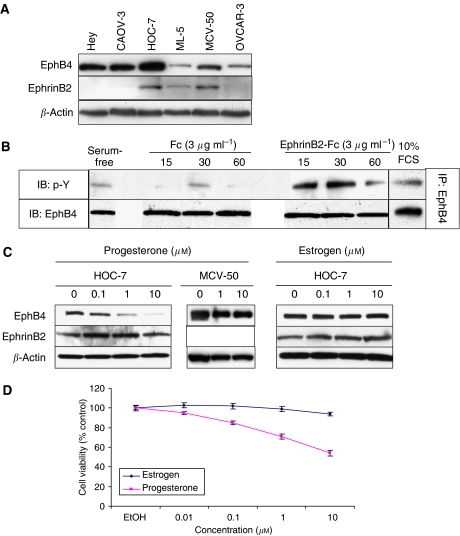
Ovarian cancer cell lines express functional EphB4 that is downregulated by progesterone. (**A**) 20 *μ*g total cell lysate from each of the ovarian cancer cell lines was run on 4–20% Tris-glycine gel and transferred to PVDF membrane. The membrane was sequentially probed with anti-EphB4, anti-EphrinB2, and anti-*β*-actin antibody. (**B**) Hey cells were serum starved overnight and stimulated for the various time periods shown with 3 *μ*g ml^−1^ clustered EphrinB2/Fc or Fc alone. EphB4 was immunoprecipitated from 100 *μ*g whole-cell lysates and phosphorylation status analysed by antiphosphotyrosine antibody immunoblotting (top row). A duplicate membrane was probed for EphB4 to document immunoprecipitation efficiency (bottom row). (**C**) Hoc-7 (ovarian cancer) and MCV-50 (ovarian cystadenoma) cells were treated with varying doses of progesterone and oestrogen for 36 h and cell lysates were analysed by immunoblotting for expression of EphB4, EphrinB2, and *β*-actin. (**D**) 1 × 10^4^ Hoc-7 cells were plated in each well of a 48-well plate and treated for 72 h with varying doses of progesterone and oestrogen. Cell viability was assessed by MTT assay and survival expressed as percentage of absorbance relative to untreated cells.

To study the biological role of EphB4, we wanted to determine the expression level of EphB4 in ovarian carcinoma cell lines in comparison with benign ovarian tumour cell lines. Western blots revealed that EphB4 was indeed expressed highly in ovarian carcinoma cell lines Hey, CAOV-3, Hoc-7, and OVCAR-3, and least in the benign ovarian cell lines MCV-50 and ML-5 (Figure 2A). Hoc-7 expressed high levels of EphB4 compared with other ovarian cell lines. EphrinB2 is expressed at negligible level to low levels in all ovarian cell lines, including the benign ovarian cell line (ML-5). An equal amount of protein loaded in each lane was confirmed by probing the same blots for *β*-actin (Figure 2A). EphB4 expressed on ovarian cancer cell lines is functional. Stimulation of Hey cells with 3 *μ*g ml^−1^ EphrinB2/Fc chimeric protein (in the absence of serum), but not Fc fragment alone, results in phosphorylation of EphB4 in 10 min, which begins to diminish by 60 min (Figure 2B). Also, addition of foetal calf serum to serum-starved Hey cells induces a small degree of EphB4 phosphorylation above baseline.

### Progesterone reduces EphB4 expression and cell growth

Menstrual hormones are known to regulate ovarian tumour development and progression (Danforth *et al*, 2007). In particular, progesterone is growth inhibitory in several previous reports (Seeger and Mueck, 2006). We were therefore interested in determining if progesterone and oestrogen affect the expression of EphB4. Hoc-7 cells express functional progesterone and oestrogen receptor. Treatment of Hoc-7 cells with progesterone led to a dose-dependent reduction in EphB4 expression, whereas oestrogen had no effect on EphB4 levels (Figure 2C). Both hormones had no effect on EphB4 expression in the benign MCV-50 cells (Figure 2C and data not shown). Progesterone, but not oestrogen, similarly inhibited EphB4 expression in Hey cells as well (data not shown). Both Hoc-7 and Hey cells treated with progesterone showed a dose-dependent reduction in cell survival. Ten micromolar progesterone resulted in nearly 50% reduction in cell survival in Hoc-7 cells (Figure 2D).

### Inhibition of EphB4 results in reduced viability

**Figure 3:**
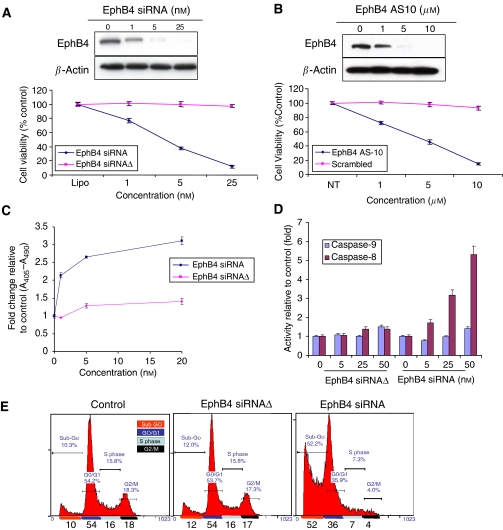
EphB4 knockdown leads to tumour cell apoptosis. (**A**) Hev cells were transiently transfected with EphB4-specific siRNA (EphB4-siRNA) and 48 h later, 20 *μ*g whole-cell lysates were analysed by immunoblotting for EphB4 and *β*-actin levels (top panel). 1 × 10^4^ Hey cells were transfected with mutated EphB4 siRNAΔ or native EphB4-siRNA and plated in a 48-well plate. Cell viability was assessed by MTT assay at 48 h and survival expressed as percentage of absorbance relative to untreated cells (bottom panel). (**B**) Hey cells were treated with varying doses of EphB4-specific ODN (AS-10). Seventy-two hours later, 20 *μ*g whole-cell lysates were analysed by immunoblotting for EphB4 and *β*-actin levels (top panel). 1 × 10^4^ Hey cells were treated with scrambled or AS-10 ODN. Cell viability was assessed by MTT assay at 72 h and survival expressed as percentage of absorbance relative to untreated cells. (**C**) Hey cells were transiently transfected with EphB4-specific siRNA (EphB4-siRNA) or mutated siRNA (EphB4-siRNAΔ). Apoptosis was analysed by ELISA for cytoplasmic nucleosomes as detailed in the Materials and Methods section using whole-cell lysates. (**D**) Caspase-8 and caspase-9 activation was assayed colorimetrically in these cells and expressed as percent activity compared with lipofectamine-treated cells. (**E**) Cell-cycle analysis of Hey cells treated with lipofectamine alone (control) or treated with 25 nM EphB4-specific siRNA (EphB4 siRNA) or mutant siRNA (EphB4 siRNAΔ) for 36 h. The percentage of cells in G_0_, G_1_, S, G_2_, and M phase are indicated. Similar results were obtained in three independent experiments.

To determine the biological function of EphB4 in ovarian cancer, we designed several siRNAs to knockdown EphB4 expression and selected the compound that best-inhibited EphB4 expression on Western blots (Figure 3A, upper panel). EphB4 siRNA lead to a corresponding dose-dependent reduction in EphB4 expression levels in Hoc-7 cells as well (data not shown). Transfection with EphB4 siRNA mutated at three bases (EphB4 siRNAΔ) or nonspecific GFP siRNA had no effect on expression levels of EphB4 (data not shown), indicating that the reduction in EphB4 expression is a specific effect of EphB4 siRNA. Concomitant with reduction in levels of EphB4 expression, EphB4 siRNA treatment, but not treatment with mutated EphB4 siRNAΔ, resulted in a dose-dependent reduction in Hey cell numbers. At a dose of 25 nM siRNA, nearly 90% cell number was reduced (Figure 3A, lower panel). The dose of siRNA to achieve 50% cell number reduction was 4 nM. Similarly, treatment of HOC-7 cells with 25 nM EphB4 siRNA resulted in 75% reduction in cell numbers (data not shown). Treatment of EphB4-negative tumour cell lines with EphB4 siRNA does not affect tumour cell numbers (Xia *et al*, 2005 and data not shown).

For use *in vivo*, we generated a panel of EphB4 antisense oligonulceotides and selected the molecule AS-10 that had maximal reduction in EphB4 levels for further studies (Figure 3B, upper panel). Similarly, treatment with 10 *μ*M AS-10, but not scrambled ODN, resulted in 90% reduction in cell numbers, with an ED_50_ of 4 *μ*M (Figure 3B, lower panel).

### Knockdown of EphB4 leads to apoptosis and activation of the death receptor caspase pathway

We were interested in determining the cause for reduced cell numbers following EphB4 knockdown. Hey cells were transfected with 25 nM EphB4 siRNA and 36 h later, subjected to cell-cycle analysis (Figure 3C). Although 90% of control cells and 88% of EphB4 siRNAΔ transfected cells entered the G_0_ phase and progressed along the cell cycle, only 48% of EphB4 siRNA-treated cells progressed into the cell cycle.

Preventing cells from entering the G_0_ phase of the cell cycle is consistent with induction of apoptosis. To further corroborate this, we quantitated cytoplasmic nucleosomes by ELISA in Hey cells 48 h after transfection with EphB4 siRNA (Figure 3D). Transfection with 25 nM EphB4 siRNA resulted in a three-fold increase in cytoplasmic nucelosomes, whereas transfection with EphB4 siRNAΔ had no effect. Apoptosis can be induced by activation of the extrinsic, membrane-originating pathway that involves casapse-8, or the intrinsic mitochondrial pathway that does not involve casapse-8. Transfection of Hey cells with 25 nM EphB4 siRNA resulted in 3.2-fold activation in caspase-8, whereas no such effect was observed with EphB4 siRNAΔ (Figure 3E). Neither the active nor the mutated siRNA resulted in appreciable casapse-9 activation. EphB4 antisense oligonucleotides induced a similar cell-cycle profile and caspase-8 activation (data not shown). Thus, EphB4 knockdown in Hey cells results in cell death owing to induction of apoptosis predominantly via the extrinsic caspase-8 pathway.

### EphB4 regulates cell migration and invasion

**Figure 4:**
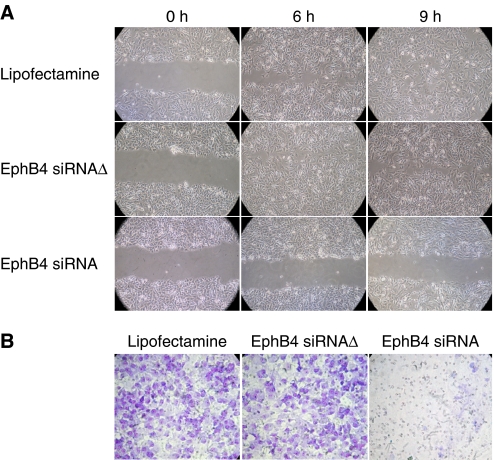
EphB4 favours tumour cell migration and invasion. (**A**) Confluent cultures of Hey cells were scraped with a plastic Pasteur pipette to produce 3-mm-wide cell-free zone in the monolayer. The ability of the cells to migrate and close the wound following transfection with 25 nM EphB4-specific (EphB4 siRNA) or mutant siRNA (EphB4 siRNAΔ) was assessed over 9 h. (**B**) Invasion of Hey cells into Matrigel-coated inserts was studied as described in the Materials and Methods section. Cells invading the underside of the inserts in response to 10 *μ*g ml^–1^ EGF in the lower chamber were fixed and stained with Giemsa. Representative photomicrographs are shown.

The higher levels of expression of EphB4 in malignant compared to benign cell lines could be suggestive of higher EphB4 levels contributing to more malignant behaviour by increasing cell migration and invasion of extracellular matrix proteins. We therefore determined if EphB4 participates in the migration of ovarian cell lines. These experiments were carried out over much shorter time periods (9–12 h), at which times no appreciable cell death was observed. Confluent Hey cultures were wounded by a single scrape with a sterile plastic Pasteur pipette, which left a 3 mm cell-free zone with clearly defined borders. Migration of cells into the cleared zone in the presence of EphB4 siRNA and EphB4 AS-10 were evaluated and quantified over 9 h. Control cells and EphB4 siRNAΔ-transfected cells migrate rapidly to cover the wound resulting in near complete wound healing by 9 h, whereas transfection of 25 nM EphB4 siRNA results in marked inhibition of cell migration and wound healing at 6 and 9 h (Figure 4A). A similar reduction in cell migration was observed with AS-10 (data not shown).

Malignant cells are capable of degrading extracellular matrix and invading through tissues. To study this function, Hey cells were cultured on Matrigel coated on the inner chamber of a Boyden double chamber for 12 h in the presence of 10*μ*g EGF in the outer chamber. Cells migrating to the under surface of the inner chamber were stained and visualised 12 h later. Control cells migrated readily to the under surface of the membrane in 12 h (Figure 4B). Whereas transfection with EphB4 siRNAΔ had no effect on cellular invasion, EphB4 siRNA nearly completely abolished cell migration across the basement membrane. A similar effect was observed with AS-10 (data not shown). Thus, EphB4 confers to ovarian cancer cells the ability to migrate and invade basement membranes, known characteristics of an aggressive malignancy.

### EphB4 antisense ODN inhibits tumour growth *in vivo*

**Figure 5:**
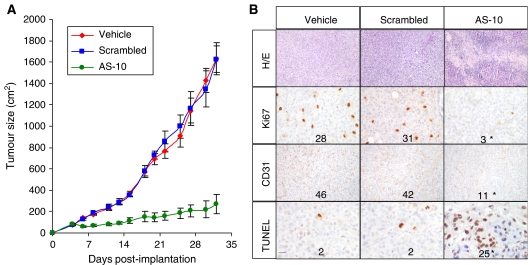
EphB4-specific antisense ODN inhibits tumour growth in a murine ovarian cancer xenograft model. (**A**) 2 × 10^6^ Hey cells were implanted in the flank of 10- to 12-week-old female Balb/C athymic mice and tumour volume measured as detailed in the Materials and Methods section. Mice were administered vehicle alone (vehicle), or 10 mg kg^–1^ scrambled ODN (scrambled) or EphB4-specific antisense ODN (AS-10) intraperitoneally daily starting day 4 after cell implantation. Animals were killed 5 weeks later and tumours harvested. (**B**) Sections (5 *μ*m) of formalin-fixed paraffin embedded sections were stained with hematoxylin/eosin and analysed by immunohistochemistry for Ki-67 and CD31 expression. Apoptosis was evaluated by TUNEL with the *in situ* apoptosis staining kit. Number of cells staining positive was averaged over five random high-power fields by a blinded observer and indicated in each micrograph. ^*^P<0.05 between AS-10 and control group. Bar in bottom left panel represents 200 *μ*m in H/E, 100 *μ*m in CD31, and 75 *μ*m in other photomicrographs.

We then studied the effect of systemic antisense administration in mice-bearing human ovarian cancer xenografts. 2 × 10^6^ Hey cells were injected in the flank of 10- to 12-week old, female Balb/C athymic mice. On day 4 after cell implantation, mice were divided randomly into three groups (*n*=6 mice per group, experiment repeated twice) and treated by intraperitoneal injection with PBS (vehicle), scrambled ODN or AS-10 at a dose of 10 mg kg^−1^. Over 5 weeks, AS-10 treatment resulted in greater than 85% smaller tumours compared with vehicle-treated tumours, whereas scrambled ODN treatment had no effect (Figure 5A). The mice appeared healthy during the entire time period, fed well and were active. Serum levels of TNF-*α*, and IL-10 and spleen weights at killed were similar between the three groups indicating that ODNs did not induce inflammatory cytokines (data not shown). Harvested tumours were evaluated by H/E (Figure 5B, upper panel), which shows large areas of tumour necrosis in AS-10-treated tumours. Immunohistochemical evaluation with Ki-67 staining shows a 10-fold reduction in proliferative cells (Figure 5B, second panel) and a 12-fold increase in TUNEL-positive cells (Figure 5B, third panel) consistent with induction of apoptosis as noted *in vitro*. Finally, AS-10 treatment resulted in 75% fewer tumour microvessels by CD31 immunostaining (Figure 5B, lower panel). Thus, systemic administration of AS-10 inhibits growth of Hey tumours in mice, along with inhibition of tumour cell proliferation, induction of apoptosis, and reduction of tumour microvasculature.

## DISCUSSION

In this study, we have evaluated the expression of EphB4 in ovarian cancer specimens using highly sensitive and specific monoclonal antibodies against the extracellular domain of EphB4. We show that EphB4 is overexpressed in a vast majority of ovarian cancers, with minimal or no expression in normal ovarian epithelium. We confirmed EphB4 expression complementarily by studying staining of sections and Western blotting of frozen samples. Overexpression of EphB4 correlates with advanced tumour stage and is associated with poor clinical outcome. A recent report by Wu *et al* (2006) has also examined mRNA and protein levels of EphB2 and EphB4 in a cohort of 115 ovarian cancer specimens. EphB4 was expressed in 80% of the tumour tissues. High expression of EphB4 on immunostaining correlated with poorer survival (*P*=0.003) and poorer response to chemotherapy (*P*=0.036). EphB4 expression may thus be a prognostic marker for outcome and response to therapy in ovarian cancer.

We were thus interested in understanding the biological role played by EphB4 in ovarian cancer. To that end, we studied several ovarian cancer cells line *in vitro.* The cell lines tested in this study replicate faithfully human tumour tissue data. All cancer cell lines examined showed high levels of EphB4 expression, whereas benign tumour cell lines showed significantly lower levels of EphB4. Normal ovarian epithelium expresses little to no EphB4. Thus, EphB4 expression is restricted to malignant ovarian disease with increased expression with advanced disease stage. Further, all cell lines examined express low levels of the ligand EphrinB2. This raises the possibility that EphB4 expressed on ovarian cells may be constitutively activated by paracrine or autocrine stimulation. Further, we found detectable basal levels of EphB4 phosphorylation under serum-free conditions in Hey cells, which do not express EphrinB2 at detectable levels on Western blot. It is thus possible that high levels of EphB4 is sufficient to result in constitutive activation of EphB4, analogous to several other receptor tyrosine kinases.

We have shown previously that overexpression of EphB4 in tumour cells is tightly regulated by known transforming agents such as EGFR (Masood *et al*, 2006) and HER-2/neu (Kumar *et al*, 2006), and inhibited by tumour suppressors such as p53 (Xia *et al*, 2006) and PTEN (Xia *et al*, 2005). Here we show that progesterone, a known tumour suppressor also regulates EphB4 expression. Hoc-7 cells are known to express functional progesterone receptor and have reduced viability in response to progesterone receptor agonists. We show for the first time that progesterone downregulates EphB4 expression in a dose-dependent fashion. Downregulation of EphB4 correlates with loss in cell viability. oestrogen, to the contrary, has no effect on EphB4 levels in Hoc-7 cells.

We then evaluated the role of EphB4 in ovarian tumour biology by achieving selective downregulation of EphB4 expression with specific siRNA and antisense ODNs. EphB4 knockdown led to a profound reduction in tumour cell survival accompanied by the induction of apoptosis. Following EphB4 knockdown, cells were unable to enter the G_0_ phase of the cell cycle and began to accumulate fragmented nucleosomes in the cytoplasm consistent with apoptosis. In addition, there was significant induction of caspase-8 activity, and a much smaller induction of caspase-9 activity, indicating that EphB4 protects tumour cells from the extrinsic, membrane-originating apoptotic pathways. Several possible mechanisms may underlie this antiapoptotic effect of EphB4. We have shown in breast and head and neck cancer cells that the extracellular domain of EphB4 is capable of protecting tumour cells from TRAIL-induced apoptosis (Kumar *et al*, 2006; Masood *et al*, 2006). Stimulation of death receptors 4 and 5 by TRAIL results in selective tumour-cell death, whereas normal cells have several mechanisms to bypass the proapoptotic effect of TRAIL. Thus, overexpression of EphB4 by tumour cells may interrupt TRAIL-mediated apoptosis thus providing survival advantage to tumour cells. In addition, we have shown that EphB4 upregulates several antiapoptotic proteins, particularly bcl-xl, in bladder and breast cancer cells (Kumar *et al*, 2006; Xia *et al*, 2006), thus suggesting other sites of action by which EphB4 may inhibit tumour cell apoptosis.

We suspected that if EphB4 expression could promote tumour cell viability, it might also promote other features of malignant carcinomas, such as tumour migration and invasion. Our human tumour data confirm that increased expression of EphB4 correlates with the presence of peritoneal disease and ascites, consistent with extra-ovarian spread of the disease. In addition, previous studies by Munarini *et al* (2002) have shown that overexpression of EphB4 in the breast of mice in the context of neuT results in highly malignant breast cancers that metastasise to the lung, a phenomenon that was uniformly absent in tumours of mice that overexpressed neuT only (Munarini *et al*, 2002). Our studies *in vitro* confirm that EphB4 knockdown leads to a profound inhibition in the ability of tumour cells to migrate and re-populate a wound or invade through basement membrane. These effects were observed at early time points when siRNA had no effect on cell survival. It is likely that inhibition of migration following knockdown of EphB4 is an early event and involves downstream pathways that may be different from those that impact cell survival. Hence, EphB4 affords a malignant phenotype to ovarian cancer cells by favouring tumour cell migration and invasion, independent of its direct prosurvival signals.

EphB4 is critically required for blood vessel maturation in the foetus (Gerety *et al*, 1999) and at adult sites of neovascularization (Shin *et al*, 2001). In addition, tumour cell-expressed EphB4 can interact with endothelial EphrinB2 to favour tumour vascularisation (Noren *et al*, 2004). Hence, apart from providing direct survival cues to tumour cells *in vitro*, EphB4 may also have a proangiogenic effect *in vivo*. Therefore, targeting EphB4 may have a dual benefit in ovarian cancer – abolishing direct tumour cell antiapoptotic signals and inhibiting tumour vascularisation. We have tested this hypothesis in a murine model of human ovarian tumour xenograft. EphB4 knockdown by systemic antisense administration led to an 85% reduction in tumour volume at 6 weeks. This was accompanied by a reduction in tumour cell proliferation and increased tumour cell apoptosis. In addition, EphB4-depleted tumours had a significant reduction in tumour microvasculature, consistent with an antiangiogenic effect of EphB4 knockdown *in vivo*.

EphB4 is highly expressed by a majority of ovarian cancers and its expression correlates with advanced disease stage, presence of ascites and decreased survival. Our study shows for the first time that the overexpressed receptor favours tumour cell survival by inhibiting apoptosis, and supports tumour cell migration and invasion. EphB4 knockdown inhibits murine tumour xenografts by inducing tumour cell apoptosis and reducing tumour microvasculature. Taken together, these results establish EphB4 as a novel target for biological therapy in ovarian cancer.
